# Crystal structure of homodinuclear platinum complex containing a metal–metal bond bridged by hydride and phosphide ligands

**DOI:** 10.1107/S205698901800868X

**Published:** 2018-06-19

**Authors:** Sakina Ouis, Djamil Azzedine Rouag, Lamia Bendjeddou, Corinne Bailly

**Affiliations:** a Unité de Recherche Chimie de l’Environnement et Moléculaire, Structurale ’CHEMS’, Faculté des Sciences Exactes, Campus Chaabet Ersas, Université Frères Mentouri Constantine 1, 25000 Constantine, Algeria; b Service de Radiocristallographie, Institut de Chimie, UMR 7177 CNRS-Université de Strasbourg, 1 Rue Blaise Pascal, 67008 Strasbourg cedex, France

**Keywords:** platinum(II) complex, hydrido bridge, phosphide ligand, crystal structure

## Abstract

In the title compound, μ-di­phenyl­phosphido-μ-hydrido-bis­[bromido­(tri­phenyl­phosphane-κ*P*)platinum(II)] diethyl ether monosolvate, [Pt_2_Br_2_(C_12_H_10_P)H(C_18_H_15_P)_2_]·C_4_H_10_O or [Pt_2_(μ-H)(μ-PPh_2_)Br_2_(PPh_3_)_2_]·(C_2_H_5_)_2_O, the Pt^II^ atoms are coordinated in a distorted square-planar arrangement, with one hydrido and one phosphido ligand bridging in a *trans* position. In the lattice, C—H⋯·O and C—H⋯π interactions are present. This complex has a total number of 32 electrons, 16 electrons for each Pt^II^ atom. One of the Br atoms is disordered over two positions in a 0.92:0.08 ratio.

## Abstract 2

The hydrido and phosphido ligands bridge the two Pt^II^ atoms to form the dinuclear complex, in which both Pt^II^ atoms are in a distorted square-planar arrangement.

## Chemical context   

Transition metal hydrides play a central role in many homogeneous catalytic reactions (Bertolasi *et al.*, 1993 ▸; Clegg *et al.*, 1996 ▸) and are very important in hydrogenation or hydro­formyl­ation. Their characterization is commonly carried out by NMR spectroscopy, X-ray analysis or neutron diffraction (Ciriano *et al.*, 1978 ▸). Hydrides of Pt^II^ are the most numerous of any transition metal hydride group (Leoni *et al.*, 1995 ▸; Bachechi *et al.*, 1993 ▸). In addition to the presence of the hydride ligand, the complexes invariably have a coordinated phosphine. Pure complexes are usually both air stable and kinetically inert (Roundhill, 1978 ▸).
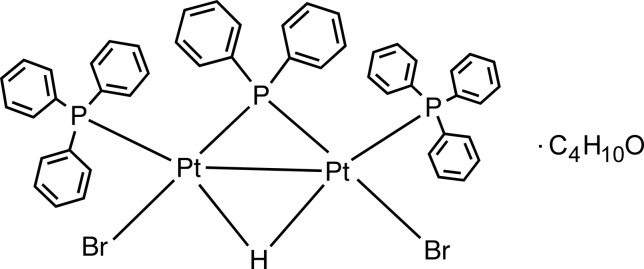


We report here the synthesis and structural analysis of a new hydrido-bridged diplatinum complex, μ-di­phenyl­phos­phido-μ-hydrido-bis­[bromido­(tri­phenyl­phosphane-κ*P*)platinum(II)] diethyl ether monosolvate. One of the attractive features of this dinuclear complex is that it is doubly bridged by hydrido and phosphido ligands in a *trans* fashion. The bridging of metal–metal-bonded homodinuclear complexes with a phosphido ligand allows the stabilization of the metal–metal bond. Many phosphido complexes (with and without a metal–metal bond) have been well documented and both their structural and reactivity features investigated (Stephan, 1989 ▸; He *et al.*, 1992 ▸; Comte *et al.*, 1997 ▸; Lavastre *et al.*, 1997 ▸; Mohamed *et al.*, 2015 ▸).

## Structural commentary   

**Figure 1:**
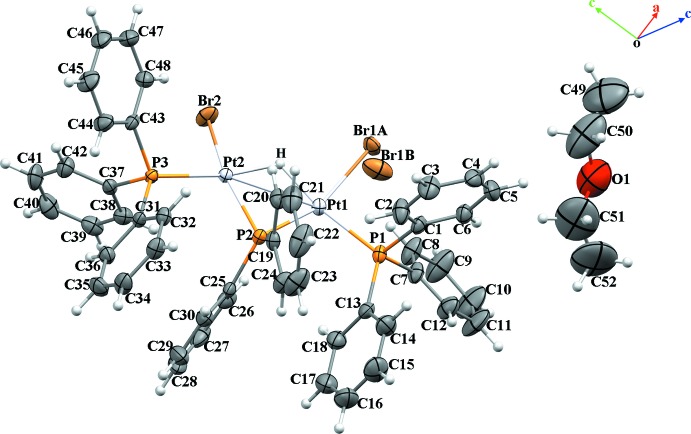
The asymmetric unit of title compound, showing the atom-numbering scheme. Displacement ellipsoids are drawn at the 50% probability level.

**Figure 2:**
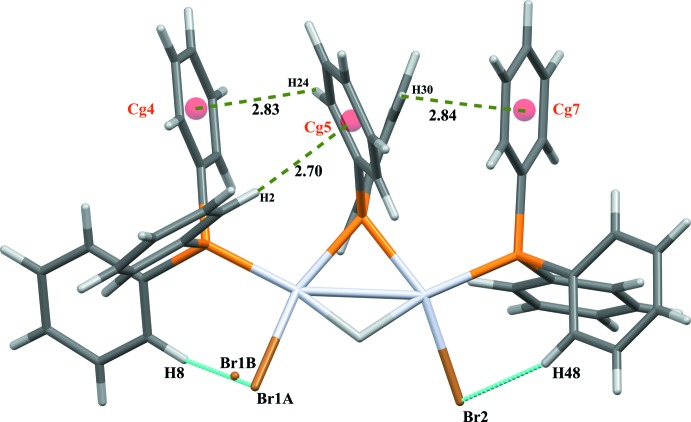
View showing intra­molecular C—H⋯Br hydrogen bonds (blue dashed lines) and C—H⋯π (green dashed lines) inter­actions (Table 2 ▸).

The asymmetric unit of the title compound consists of an organometallic mol­ecule which is a neutral dinuclear Pt^II^ complex, [Pt_2_(μ-H)(μ-PPh_2_)Br_2_(PPh_3_)_2_], and one solvent ether mol­ecule (Fig. 1 ▸). The dinuclear complex is composed of two triangles formed by two platinum atoms and one phospho­rus (P2), and by two platinum and one hydrogen atom. The coordination sphere of each platinum atom is completed by a terminal phosphine (P1, P3) and two bromides (Br1, Br2). The two platinum atoms are linked by phosphido and hydrido bridges and a formal metal–metal bond. The Pt—Pt bond increases from 2.667 (3) Å in the starting complex to 2.8365 (4) Å in the title complex because of the difference in bridging atoms and probably also because of steric hindrance between the phenyl groups of the tri­phenyl­phosphine unit and repulsion between the triphenylphosphine groups and the bromide atoms. The value of this bond length is comparable to those in dinuclear phosphido- and hydrido-bridged compounds (2.58–2.9 Å; Jans *et al.*, 1983 ▸; Bender *et al.*, 1999 ▸; Archambault *et al.*, 2001 ▸) but is one of the longest. The angle of the phosphido bridge, Pt1—P2—Pt2, is 79.24 (3)° because of the long Pt—Pt bond and is one of the largest phosphido bridge angles observed (Bachechi *et al.*, 1983 ▸). The platinum atoms are coordinated in a distorted square-planar arrangement with one hydrido and one phosphido ligand in a bridging *trans* position. The phospho­rus atoms P1 and P3 are located in the same plane as atoms Pt1/P2/Pt2 with the bromide atoms displaced by 0.0117 (3) and 0.0250 (2) Å from the plane. The Pt1—P1 and Pt2—P3 bond lengths [2.2616 (13) and 2.2731 (11) Å, respectively] are comparable, and consistent with literature values for a platinum–phospho­rus bond. The Pt—Br bonds [2.5023 (5) and 2.5084 (6) Å; Table 1 ▸] are slightly longer than those found in the literature (Bachechi *et al.*, 1983 ▸). Intra­molecular C—H⋯Br hydrogen bonds and C—H⋯π (Table 2 ▸, Fig. 2 ▸) inter­actions are observed.

## Supra­molecular features   

**Figure 3:**
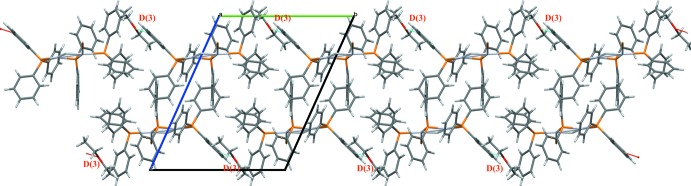
Partial packing diagram of the molecules along the *b*-axis direction, showing the C—H⋯O hydrogen bonds (Table 2 ▸).

As shown in Fig. 3 ▸, C45—H45⋯O1^i^ hydrogen bonds (Table 2 ▸, ) link the complex and solvent mol­ecules.

## NMR Spectroscopy   

**Figure 4:**
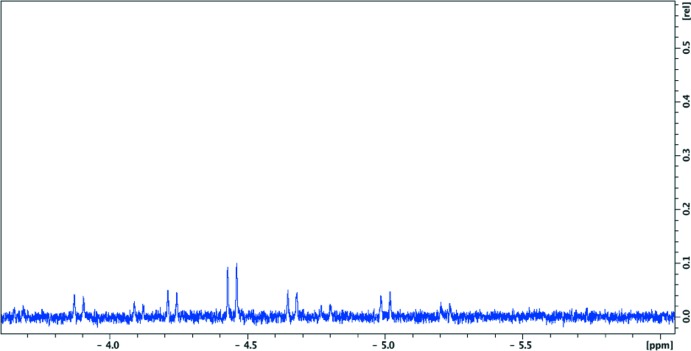
Part of the ^1^H NMR spectrum of the hydride bridging absorbing at −4.55 ppm.

The ^1^H NMR spectrum was recorded on a FT Bruker AVANCE III instrument at 296 K. The spectrum exhibits high field resonance, which unequivocally indicates the presence of a bridging hydride ligand absorbing at −4.5 ppm (Fig. 4 ▸) with ^1^ *J* Pt—H 445 Hz, ^2^ *J* P2—H 13Hz, ^2^ *J* P1—H 87 Hz, identical to ^2^ *J* P3—H (Jans *et al.*, 1983 ▸).

## Database survey   

A search of the Cambridge Structural Database (Version 5.38; Groom *et al.*, 2016 ▸) gave 60 hits for a direct platinum platinum bond with a hydrido bridge. Only one contains the same core as the title compound with hydrido and phosphido bridges (Jans *et al.*, 1983 ▸).

## Synthesis and crystallization   

In a 100ml schlenk, [Pt_2_(μ-*o*-C_6_H_4_PPh_2_)(PPh_3_)_2_] (135 mg, 0.1 mmol) in freshly distilled THF (30 ml) was stirred at room temperature for three h with a 2*M* solution of HBr (230 µl, 0.2 mmol). The product, a yellow powder, was recrystallized from ether/pentane (*v*:*v* = 1:1) yielding pale-yellow crystals.

## Refinement   

Crystal data, data collection and structure refinement details are summarized in Table 3 ▸. The hydrido H atom was located in difference-Fourier maps and refined with a distance restraint of 1.7 Å using the command DFIX in *SHELXL2014*. C-bound H atoms were positioned geometrically and refined as riding atoms, with C—H = 0.95 (aromatic), 0.99 (CH_2_) and 0.98 Å (CH_3_) and with *U*_iso_(H) = 1.2*U*_eq_(C) or 1.5*U*_eq_(Cmeth­yl). Atom Br1 is disordered over two positions in a 0.92:0.08 ratio.

## Supplementary Material

Crystal structure: contains datablock(s) global, I. DOI: 10.1107/S205698901800868X/xu5929sup1.cif

Structure factors: contains datablock(s) I. DOI: 10.1107/S205698901800868X/xu5929Isup2.hkl

CCDC reference: 1849017

Additional supporting information:  crystallographic information; 3D view; checkCIF report

## Figures and Tables

**Table 1:** Selected geometric parameters (Å, °)

Pt1—Pt2	2.8365 (4)	Pt1—P2	2.2217 (10)
Pt1—Br1*A*	2.5084 (6)	Pt2—Br2	2.5023 (5)
Pt1—Br1*B*	2.364 (9)	Pt2—P2	2.2266 (11)
Pt1—P1	2.2616 (13)	Pt2—P3	2.2731 (11)
			
Pt2—Pt1—Br1*A*	112.09 (2)	Pt1—Pt2—P3	154.61 (3)
Pt2—Pt1—Br1*B*	124.3 (3)	Br2—Pt2—P2	157.93 (3)
Pt2—Pt1—P1	155.02 (3)	Br2—Pt2—P3	93.56 (3)
Pt2—Pt1—P2	50.46 (3)	P2—Pt2—P3	104.41 (4)
Br1*A*—Pt1—P1	91.84 (3)	P1—Pt1—H	169.7 (10)
Br1*A*—Pt1—P2	158.53 (3)	P2—Pt1—H	85.5 (8)
Br1*B*—Pt1—P1	80.7 (3)	Pt2—Pt1—H	35.1 (9)
Br1*B*—Pt1—P2	174.7 (3)	Br2—Pt2—H	77.7 (11)
P1—Pt1—P2	104.59 (4)	P2—Pt2—H	85.4 (11)
Pt1—Pt2—Br2	111.58 (1)	P3—Pt2—H	169.6 (12)
Pt1—Pt2—P2	50.31 (3)	Pt1—P2—Pt2	79.24 (3)

**Table 2:** Hydrogen-bond geometry (Å, °) *Cg*4, *Cg*5 and *Cg*7 are the centroids of the C13–C18, C19–C24 and C31–C36 rings, respectively.

*D*—H⋯*A*	*D*—H	H⋯*A*	*D*⋯*A*	*D*—H⋯*A*
C8—H8⋯Br1*A*	0.95	2.73	3.460 (6)	134
C8—H8⋯Br1*B*	0.95	1.99	2.719 (13)	132
C45—H45⋯O1^i^	0.95	2.54	3.484 (8)	170
C48—H48⋯Br2	0.95	2.65	3.520 (5)	153
C2—H2⋯*Cg*5	0.95	2.70	3.637 (6)	168
C24—H24⋯*Cg*4	0.95	2.83	3.459 (9)	125
C30—H30⋯*Cg*7	0.95	2.84	3.340 (5)	114

Symmetry code: (i) 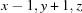.

**Table 3:** Experimental details

Crystal data	
Chemical formula	[Pt_2_Br_2_(C_12_H_10_P)H(C_18_H_15_P)_2_]·C_4_H_10_O
*M*_r_	1334.83
Crystal system, space group	Triclinic, *P* 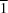
Temperature (K)	173
*a*, *b*, *c* (Å)	11.0257 (5), 14.0341 (6), 17.4469 (7)
α, β, γ (°)	113.411 (1), 94.083 (1), 96.681 (1)
*V* (Å^3^)	2440.09 (18)
*Z*	2
Radiation type	Mo *K*α
μ (mm^−1^)	7.50
Crystal size (mm)	0.25 × 0.16 × 0.08

Data collection	
Diffractometer	Bruker APEXII CCD
Absorption correction	Multi-scan (*SADABS*; Bruker, 2006 ▸)
*T*_min_, *T*_max_	0.512, 0.747
No. of measured, independent and observed [*I* > 2σ(*I*)] reflections	47421, 9969, 8359
*R*_int_	0.033
(sin θ/λ)_max_ (Å^−1^)	0.625

Refinement	
*R*[*F* ^2^ > 2σ(*F* ^2^)], *wR*(*F* ^2^), *S*	0.023, 0.057, 1.02
No. of reflections	9969
No. of parameters	556
No. of restraints	23
H-atom treatment	H atoms treated by a mixture of independent and constrained refinement
Δρ_max_, Δρ_min_ (e Å^−3^)	2.00, −1.01

Computer programs: *APEX2* and *SAINT* (Bruker, 2006 ▸), *SHELXS97* and *SHELXL97* (Sheldrick, 2008 ▸), *ORTEP-3 for Windows* (Farrugia, 2012 ▸), *OLEX2* (Dolomanov *et al.*, 2009 ▸) and *Mercury* (Macrae *et al.*, 2006 ▸).

## supplementary crystallographic information

### Crystal data

**Table:**

[Pt_2_Br_2_(C_12_H_10_P)H(C_18_H_15_P)_2_]·C_4_H_10_O	*Z* = 2
*M**_r_* = 1334.83	*F*(000) = 1284
Triclinic, *P*1	*D*_x_ = 1.817 Mg m^−^^3^
Hall symbol: -P 1	Mo *K*α radiation, λ = 0.71073 Å
*a* = 11.0257 (5) Å	Cell parameters from 9992 reflections
*b* = 14.0341 (6) Å	θ = 2.3–35.0°
*c* = 17.4469 (7) Å	µ = 7.50 mm^−^^1^
α = 113.411 (1)°	*T* = 173 K
β = 94.083 (1)°	Plate, yellow
γ = 96.681 (1)°	0.25 × 0.16 × 0.08 mm
*V* = 2440.09 (18) Å^3^	

### Data collection

**Table:**

Bruker APEXII CCD diffractometer	9969 independent reflections
Radiation source: fine-focus sealed tube	8359 reflections with *I* > 2σ(*I*)
Graphite monochromator	*R*_int_ = 0.033
Detector resolution: 18.4 pixels mm^-1^	θ_max_ = 26.4°, θ_min_ = 1.6°
φ and ω scans	*h* = −13→13
Absorption correction: multi-scan (SADABS; Bruker, 2006)	*k* = −17→17
*T*_min_ = 0.512, *T*_max_ = 0.747	*l* = −21→21
47421 measured reflections	

### Refinement

**Table:**

Refinement on *F*^2^	Primary atom site location: structure-invariant direct methods
Least-squares matrix: full	Hydrogen site location: mixed
*R*[*F*^2^ > 2σ(*F*^2^)] = 0.023	H atoms treated by a mixture of independent and constrained refinement
*wR*(*F*^2^) = 0.057	*w* = 1/[σ^2^(*F*_o_^2^) + (0.0283*P*)^2^ + 3.5779*P*] where *P* = (*F*_o_^2^ + 2*F*_c_^2^)/3
*S* = 1.02	(Δ/σ)_max_ = 0.003
9969 reflections	Δρ_max_ = 2.00 e Å^−^^3^
556 parameters	Δρ_min_ = −1.01 e Å^−^^3^
23 restraints	

### Special details

**Table:**

Geometry. Bond distances, angles etc. have been calculated using the rounded fractional coordinates. All su's are estimated from the variances of the (full) variance-covariance matrix. The cell esds are taken into account in the estimation of distances, angles and torsion angles
Refinement. Refinement of F^2^ against ALL reflections. The weighted R-factor wR and goodness of fit S are based on F^2^, conventional R-factors R are based on F, with F set to zero for negative F^2^. The threshold expression of F^2^>2sigma(F^2^) is used only for calculating R-factors(gt) etc. and is not relevant to the choice of reflections for refinement. R-factors based on F^2^ are statistically about twice as large as those based on F, and R- factors based on ALL data will be even larger.

### Fractional atomic coordinates and isotropic or equivalent isotropic displacement parameters (Å^2^)

**Table:**

	*x*	*y*	*z*	*U*_iso_*/*U*_eq_	Occ. (<1)
Pt1	0.00394 (2)	0.88526 (2)	0.75388 (2)	0.0191 (1)	
Pt2	−0.06369 (2)	1.06252 (2)	0.73179 (2)	0.0177 (1)	
Br1A	0.21974 (5)	0.92152 (5)	0.82617 (4)	0.0298 (2)	0.920
Br1B	0.2082 (8)	0.8680 (9)	0.7906 (5)	0.054 (3)	0.080
Br2	0.11894 (4)	1.17492 (3)	0.71718 (3)	0.0354 (1)	
P1	−0.03201 (10)	0.72696 (8)	0.76059 (7)	0.0225 (3)	
P2	−0.18132 (9)	0.91642 (7)	0.72085 (6)	0.0184 (3)	
P3	−0.19935 (9)	1.16206 (7)	0.70978 (6)	0.0173 (3)	
C1	−0.0144 (4)	0.7457 (3)	0.8704 (3)	0.0256 (12)	
O1	0.4696 (5)	0.5756 (5)	0.9171 (4)	0.106 (3)	
C2	−0.0902 (5)	0.8066 (4)	0.9231 (3)	0.0404 (17)	
C3	−0.0777 (5)	0.8264 (4)	1.0081 (3)	0.0439 (19)	
C4	0.0081 (5)	0.7842 (4)	1.0401 (3)	0.0373 (14)	
C5	0.0852 (5)	0.7255 (4)	0.9884 (3)	0.0388 (17)	
C6	0.0748 (4)	0.7059 (3)	0.9034 (3)	0.0304 (12)	
C7	0.0683 (4)	0.6318 (3)	0.7082 (3)	0.0300 (14)	
C8	0.1758 (5)	0.6624 (4)	0.6809 (3)	0.0457 (17)	
C9	0.2501 (6)	0.5881 (5)	0.6409 (4)	0.066 (2)	
C10	0.2166 (6)	0.4845 (5)	0.6282 (5)	0.074 (3)	
C11	0.1111 (6)	0.4548 (4)	0.6580 (4)	0.064 (2)	
C12	0.0373 (5)	0.5283 (4)	0.6979 (4)	0.0444 (19)	
C13	−0.1830 (4)	0.6475 (3)	0.7150 (3)	0.0264 (12)	
C14	−0.2595 (5)	0.6088 (4)	0.7591 (3)	0.0405 (17)	
C15	−0.3708 (5)	0.5458 (4)	0.7194 (4)	0.0527 (19)	
C16	−0.4070 (5)	0.5213 (4)	0.6361 (4)	0.0528 (19)	
C17	−0.3310 (5)	0.5596 (4)	0.5912 (3)	0.0480 (17)	
C18	−0.2199 (5)	0.6222 (4)	0.6302 (3)	0.0357 (14)	
C19	−0.2826 (4)	0.9126 (3)	0.7973 (3)	0.0230 (12)	
C20	−0.2545 (4)	0.9915 (3)	0.8785 (3)	0.0308 (14)	
C21	−0.3228 (5)	0.9889 (4)	0.9415 (3)	0.0456 (19)	
C22	−0.4174 (6)	0.9072 (5)	0.9240 (4)	0.057 (2)	
C23	−0.4453 (5)	0.8285 (4)	0.8448 (4)	0.0523 (19)	
C24	−0.3775 (4)	0.8291 (4)	0.7810 (3)	0.0342 (14)	
C25	−0.2685 (4)	0.8502 (3)	0.6165 (3)	0.0235 (11)	
C26	−0.2012 (4)	0.8197 (3)	0.5484 (3)	0.0313 (14)	
C27	−0.2604 (6)	0.7795 (3)	0.4669 (3)	0.0435 (16)	
C28	−0.3867 (6)	0.7657 (4)	0.4527 (3)	0.0490 (19)	
C29	−0.4536 (5)	0.7955 (4)	0.5183 (3)	0.0486 (17)	
C30	−0.3959 (4)	0.8393 (3)	0.6011 (3)	0.0332 (14)	
C31	−0.3612 (3)	1.1109 (3)	0.6999 (2)	0.0190 (11)	
C32	−0.4044 (4)	1.1002 (3)	0.7699 (3)	0.0247 (12)	
C33	−0.5269 (4)	1.0618 (3)	0.7667 (3)	0.0298 (14)	
C34	−0.6077 (4)	1.0352 (4)	0.6945 (3)	0.0350 (16)	
C35	−0.5661 (4)	1.0467 (4)	0.6258 (3)	0.0358 (16)	
C36	−0.4429 (4)	1.0840 (3)	0.6279 (3)	0.0271 (12)	
C37	−0.1800 (3)	1.1791 (3)	0.6131 (2)	0.0207 (11)	
C38	−0.1460 (4)	1.0968 (3)	0.5462 (3)	0.0274 (12)	
C39	−0.1318 (4)	1.1054 (4)	0.4710 (3)	0.0323 (14)	
C40	−0.1494 (5)	1.1954 (4)	0.4621 (3)	0.0428 (17)	
C41	−0.1827 (5)	1.2783 (4)	0.5284 (3)	0.0441 (17)	
C42	−0.1988 (4)	1.2703 (3)	0.6036 (3)	0.0319 (14)	
C43	−0.1864 (4)	1.2956 (3)	0.7920 (2)	0.0216 (11)	
C44	−0.2896 (4)	1.3424 (3)	0.8157 (3)	0.0337 (14)	
C45	−0.2768 (5)	1.4447 (4)	0.8766 (3)	0.0400 (16)	
C46	−0.1624 (5)	1.5005 (3)	0.9142 (3)	0.0387 (16)	
C47	−0.0599 (5)	1.4555 (3)	0.8906 (3)	0.0361 (16)	
C48	−0.0707 (4)	1.3532 (3)	0.8298 (3)	0.0284 (12)	
C49	0.6136 (11)	0.7134 (8)	1.0225 (7)	0.144 (5)	
C50	0.4824 (10)	0.6726 (8)	0.9905 (7)	0.127 (4)	
C51	0.3504 (9)	0.5283 (10)	0.8798 (9)	0.150 (6)	
C52	0.3495 (10)	0.4253 (9)	0.8167 (9)	0.166 (6)	
H	0.058 (2)	1.008 (2)	0.757 (3)	0.064 (17)*	
H2	−0.15090	0.83502	0.90092	0.0480*	
H3	−0.12886	0.86917	1.04408	0.0530*	
H4	0.01445	0.79538	1.09771	0.0450*	
H5	0.14633	0.69810	1.01109	0.0460*	
H6	0.12858	0.66551	0.86829	0.0370*	
H8	0.19854	0.73368	0.68951	0.0550*	
H9	0.32364	0.60878	0.62238	0.0790*	
H10	0.26555	0.43337	0.59907	0.0880*	
H11	0.08977	0.38394	0.65086	0.0770*	
H12	−0.03455	0.50790	0.71826	0.0530*	
H14	−0.23521	0.62555	0.81695	0.0480*	
H15	−0.42267	0.51926	0.75006	0.0630*	
H16	−0.48385	0.47832	0.60909	0.0630*	
H17	−0.35586	0.54239	0.53344	0.0570*	
H18	−0.16815	0.64833	0.59923	0.0420*	
H20	−0.18837	1.04732	0.89073	0.0370*	
H21	−0.30442	1.04335	0.99663	0.0540*	
H22	−0.46376	0.90550	0.96741	0.0680*	
H23	−0.51153	0.77308	0.83347	0.0620*	
H24	−0.39550	0.77317	0.72651	0.0410*	
H26	−0.11406	0.82651	0.55799	0.0370*	
H27	−0.21389	0.76136	0.42076	0.0520*	
H28	−0.42742	0.73516	0.39663	0.0590*	
H29	−0.54077	0.78634	0.50771	0.0580*	
H30	−0.44333	0.86164	0.64663	0.0400*	
H32	−0.34940	1.11930	0.81995	0.0300*	
H33	−0.55547	1.05384	0.81427	0.0360*	
H34	−0.69183	1.00892	0.69241	0.0420*	
H35	−0.62203	1.02895	0.57642	0.0430*	
H36	−0.41483	1.09099	0.57992	0.0330*	
H38	−0.13253	1.03428	0.55205	0.0330*	
H39	−0.10969	1.04836	0.42526	0.0390*	
H40	−0.13870	1.20118	0.41047	0.0510*	
H41	−0.19453	1.34094	0.52218	0.0530*	
H42	−0.22263	1.32697	0.64873	0.0390*	
H44	−0.36933	1.30437	0.79025	0.0410*	
H45	−0.34801	1.47626	0.89230	0.0480*	
H46	−0.15438	1.57012	0.95631	0.0460*	
H47	0.01945	1.49446	0.91618	0.0430*	
H48	0.00111	1.32272	0.81398	0.0340*	
H49A	0.66194	0.69628	0.97512	0.2160*	
H49B	0.64175	0.68122	1.05981	0.2160*	
H49C	0.62444	0.78990	1.05398	0.2160*	
H50A	0.43940	0.66091	1.03469	0.1520*	
H50B	0.44406	0.72478	0.97621	0.1520*	
H51A	0.31386	0.57156	0.85407	0.1800*	
H51B	0.29961	0.52402	0.92323	0.1800*	
H52A	0.40964	0.42814	0.77859	0.2490*	
H52B	0.26718	0.39793	0.78456	0.2490*	
H52C	0.37106	0.37889	0.84346	0.2490*	

### Atomic displacement parameters (Å^2^)

**Table:**

	*U*^11^	*U*^22^	*U*^33^	*U*^12^	*U*^13^	*U*^23^
Pt1	0.0181 (1)	0.0218 (1)	0.0213 (1)	0.0054 (1)	0.0053 (1)	0.0117 (1)
Pt2	0.0161 (1)	0.0179 (1)	0.0205 (1)	0.0030 (1)	0.0034 (1)	0.0090 (1)
Br1A	0.0185 (2)	0.0384 (3)	0.0356 (4)	0.0010 (2)	−0.0020 (2)	0.0201 (3)
Br1B	0.040 (4)	0.077 (6)	0.038 (5)	−0.012 (5)	0.004 (4)	0.022 (4)
Br2	0.0235 (2)	0.0294 (2)	0.0564 (3)	0.0019 (2)	0.0144 (2)	0.0199 (2)
P1	0.0285 (6)	0.0227 (5)	0.0219 (5)	0.0102 (4)	0.0067 (4)	0.0127 (4)
P2	0.0184 (5)	0.0174 (5)	0.0214 (5)	0.0041 (4)	0.0044 (4)	0.0095 (4)
P3	0.0179 (5)	0.0155 (4)	0.0192 (5)	0.0015 (4)	0.0027 (4)	0.0080 (4)
C1	0.035 (2)	0.022 (2)	0.025 (2)	0.0053 (17)	0.0061 (18)	0.0143 (18)
O1	0.085 (4)	0.099 (4)	0.147 (6)	0.030 (3)	0.041 (4)	0.054 (4)
C2	0.058 (3)	0.044 (3)	0.030 (3)	0.026 (2)	0.014 (2)	0.020 (2)
C3	0.066 (4)	0.041 (3)	0.026 (3)	0.019 (3)	0.014 (2)	0.011 (2)
C4	0.057 (3)	0.031 (2)	0.021 (2)	−0.002 (2)	−0.001 (2)	0.011 (2)
C5	0.047 (3)	0.038 (3)	0.034 (3)	0.005 (2)	−0.007 (2)	0.020 (2)
C6	0.035 (2)	0.030 (2)	0.031 (2)	0.0066 (19)	0.004 (2)	0.017 (2)
C7	0.040 (3)	0.026 (2)	0.028 (2)	0.0175 (19)	0.011 (2)	0.0109 (19)
C8	0.062 (3)	0.036 (3)	0.054 (3)	0.022 (2)	0.032 (3)	0.026 (3)
C9	0.072 (4)	0.048 (3)	0.096 (5)	0.029 (3)	0.056 (4)	0.036 (4)
C10	0.082 (5)	0.043 (3)	0.104 (6)	0.036 (3)	0.051 (4)	0.025 (4)
C11	0.065 (4)	0.027 (3)	0.102 (5)	0.020 (3)	0.031 (4)	0.022 (3)
C12	0.037 (3)	0.032 (3)	0.066 (4)	0.012 (2)	0.013 (3)	0.019 (3)
C13	0.032 (2)	0.018 (2)	0.029 (2)	0.0038 (17)	0.0044 (18)	0.0095 (18)
C14	0.040 (3)	0.042 (3)	0.045 (3)	−0.002 (2)	0.005 (2)	0.026 (2)
C15	0.044 (3)	0.056 (3)	0.069 (4)	−0.002 (3)	0.011 (3)	0.039 (3)
C16	0.041 (3)	0.043 (3)	0.070 (4)	−0.005 (2)	−0.006 (3)	0.024 (3)
C17	0.057 (3)	0.039 (3)	0.041 (3)	0.003 (2)	−0.009 (3)	0.013 (2)
C18	0.045 (3)	0.031 (2)	0.029 (2)	0.005 (2)	0.002 (2)	0.011 (2)
C19	0.021 (2)	0.028 (2)	0.027 (2)	0.0103 (16)	0.0091 (17)	0.0159 (18)
C20	0.035 (3)	0.032 (2)	0.029 (2)	0.0123 (19)	0.006 (2)	0.014 (2)
C21	0.062 (4)	0.050 (3)	0.035 (3)	0.030 (3)	0.021 (3)	0.020 (3)
C22	0.075 (4)	0.059 (4)	0.064 (4)	0.034 (3)	0.049 (3)	0.041 (3)
C23	0.050 (3)	0.045 (3)	0.077 (4)	0.011 (3)	0.036 (3)	0.035 (3)
C24	0.031 (2)	0.032 (2)	0.046 (3)	0.0077 (19)	0.016 (2)	0.020 (2)
C25	0.033 (2)	0.0158 (18)	0.024 (2)	0.0038 (16)	0.0009 (17)	0.0110 (17)
C26	0.042 (3)	0.023 (2)	0.030 (2)	0.0040 (19)	0.009 (2)	0.0117 (19)
C27	0.082 (4)	0.024 (2)	0.025 (2)	0.012 (2)	0.010 (3)	0.009 (2)
C28	0.077 (4)	0.032 (3)	0.029 (3)	0.015 (3)	−0.015 (3)	0.005 (2)
C29	0.049 (3)	0.039 (3)	0.047 (3)	0.009 (2)	−0.019 (3)	0.010 (3)
C30	0.033 (2)	0.025 (2)	0.037 (3)	0.0057 (18)	−0.004 (2)	0.009 (2)
C31	0.0136 (18)	0.0187 (18)	0.028 (2)	0.0033 (15)	0.0039 (16)	0.0126 (17)
C32	0.027 (2)	0.024 (2)	0.026 (2)	0.0085 (17)	0.0064 (18)	0.0116 (18)
C33	0.026 (2)	0.033 (2)	0.039 (3)	0.0084 (18)	0.015 (2)	0.021 (2)
C34	0.020 (2)	0.038 (3)	0.055 (3)	0.0075 (19)	0.008 (2)	0.026 (2)
C35	0.024 (2)	0.043 (3)	0.041 (3)	0.002 (2)	−0.004 (2)	0.020 (2)
C36	0.024 (2)	0.030 (2)	0.030 (2)	0.0053 (17)	0.0010 (18)	0.0151 (19)
C37	0.0175 (19)	0.025 (2)	0.023 (2)	0.0005 (15)	0.0025 (16)	0.0143 (17)
C38	0.027 (2)	0.029 (2)	0.025 (2)	0.0015 (17)	0.0047 (18)	0.0103 (19)
C39	0.029 (2)	0.043 (3)	0.023 (2)	0.002 (2)	0.0065 (18)	0.012 (2)
C40	0.043 (3)	0.066 (3)	0.032 (3)	0.007 (3)	0.008 (2)	0.033 (3)
C41	0.058 (3)	0.047 (3)	0.045 (3)	0.013 (3)	0.007 (3)	0.036 (3)
C42	0.038 (3)	0.030 (2)	0.033 (2)	0.009 (2)	0.008 (2)	0.017 (2)
C43	0.032 (2)	0.0154 (18)	0.019 (2)	0.0055 (16)	0.0052 (17)	0.0080 (16)
C44	0.032 (2)	0.024 (2)	0.040 (3)	0.0072 (19)	0.005 (2)	0.007 (2)
C45	0.043 (3)	0.028 (2)	0.046 (3)	0.015 (2)	0.014 (2)	0.008 (2)
C46	0.056 (3)	0.021 (2)	0.032 (3)	0.010 (2)	0.002 (2)	0.003 (2)
C47	0.043 (3)	0.024 (2)	0.035 (3)	−0.001 (2)	−0.005 (2)	0.009 (2)
C48	0.031 (2)	0.026 (2)	0.026 (2)	0.0035 (18)	0.0010 (18)	0.0091 (19)
C49	0.157 (9)	0.097 (7)	0.145 (10)	0.033 (7)	0.043 (8)	0.007 (7)
C50	0.140 (7)	0.100 (6)	0.170 (9)	0.056 (6)	0.102 (7)	0.062 (6)
C51	0.054 (5)	0.175 (10)	0.239 (14)	0.011 (6)	0.044 (6)	0.102 (9)
C52	0.085 (7)	0.139 (9)	0.257 (15)	−0.049 (7)	−0.013 (8)	0.088 (9)

### Geometric parameters (Å, º)

**Table:**

Pt1—Pt2	2.8365 (4)	C40—C41	1.385 (7)
Pt1—Br1A	2.5084 (6)	C41—C42	1.382 (7)
Pt1—Br1B	2.364 (9)	C43—C48	1.390 (6)
Pt1—P1	2.2616 (13)	C43—C44	1.386 (6)
Pt1—P2	2.2217 (10)	C44—C45	1.389 (7)
Pt2—Br2	2.5023 (5)	C45—C46	1.371 (8)
Pt2—P2	2.2266 (11)	C46—C47	1.368 (8)
Pt2—P3	2.2731 (11)	C47—C48	1.390 (7)
Pt1—H	1.73 (3)	C2—H2	0.9500
Pt2—H	1.73 (3)	C3—H3	0.9500
P1—C13	1.823 (5)	C4—H4	0.9500
P1—C1	1.820 (5)	C5—H5	0.9500
P1—C7	1.833 (5)	C6—H6	0.9500
P2—C25	1.817 (5)	C8—H8	0.9500
P2—C19	1.812 (5)	C9—H9	0.9500
P3—C37	1.819 (4)	C10—H10	0.9500
P3—C31	1.814 (4)	C11—H11	0.9500
P3—C43	1.834 (4)	C12—H12	0.9500
C1—C6	1.383 (6)	C14—H14	0.9500
C1—C2	1.388 (7)	C15—H15	0.9500
O1—C50	1.434 (13)	C16—H16	0.9500
O1—C51	1.387 (13)	C17—H17	0.9500
C2—C3	1.389 (7)	C18—H18	0.9500
C3—C4	1.369 (8)	C20—H20	0.9500
C4—C5	1.377 (8)	C21—H21	0.9500
C5—C6	1.390 (7)	C22—H22	0.9500
C7—C12	1.388 (8)	C23—H23	0.9500
C7—C8	1.393 (7)	C24—H24	0.9500
C8—C9	1.396 (9)	C26—H26	0.9500
C9—C10	1.381 (11)	C27—H27	0.9500
C10—C11	1.394 (10)	C28—H28	0.9500
C11—C12	1.384 (9)	C29—H29	0.9500
C13—C18	1.394 (7)	C30—H30	0.9500
C13—C14	1.384 (7)	C32—H32	0.9500
C14—C15	1.381 (8)	C33—H33	0.9500
C15—C16	1.370 (9)	C34—H34	0.9500
C16—C17	1.389 (8)	C35—H35	0.9500
C17—C18	1.375 (8)	C36—H36	0.9500
C19—C24	1.399 (7)	C38—H38	0.9500
C19—C20	1.393 (7)	C39—H39	0.9500
C20—C21	1.386 (7)	C40—H40	0.9500
C21—C22	1.376 (9)	C41—H41	0.9500
C22—C23	1.368 (9)	C42—H42	0.9500
C23—C24	1.387 (8)	C44—H44	0.9500
C25—C26	1.393 (7)	C45—H45	0.9500
C25—C30	1.390 (6)	C46—H46	0.9500
C26—C27	1.382 (7)	C47—H47	0.9500
C27—C28	1.374 (9)	C48—H48	0.9500
C28—C29	1.357 (8)	C49—C50	1.473 (16)
C29—C30	1.394 (7)	C51—C52	1.43 (2)
C31—C32	1.398 (6)	C49—H49A	0.9800
C31—C36	1.384 (6)	C49—H49B	0.9800
C32—C33	1.383 (6)	C49—H49C	0.9800
C33—C34	1.382 (7)	C50—H50A	0.9900
C34—C35	1.374 (7)	C50—H50B	0.9900
C35—C36	1.390 (6)	C51—H51A	0.9900
C37—C38	1.389 (6)	C51—H51B	0.9900
C37—C42	1.391 (6)	C52—H52A	0.9800
C38—C39	1.382 (7)	C52—H52B	0.9800
C39—C40	1.366 (8)	C52—H52C	0.9800
			
Pt2—Pt1—Br1A	112.09 (2)	C43—C48—C47	120.1 (4)
Pt2—Pt1—Br1B	124.3 (3)	C1—C2—H2	120.00
Pt2—Pt1—P1	155.02 (3)	C3—C2—H2	120.00
Pt2—Pt1—P2	50.46 (3)	C4—C3—H3	120.00
Br1A—Pt1—P1	91.84 (3)	C2—C3—H3	120.00
Br1A—Pt1—P2	158.53 (3)	C5—C4—H4	120.00
Br1B—Pt1—P1	80.7 (3)	C3—C4—H4	120.00
Br1B—Pt1—P2	174.7 (3)	C4—C5—H5	120.00
P1—Pt1—P2	104.59 (4)	C6—C5—H5	120.00
Pt1—Pt2—Br2	111.58 (1)	C1—C6—H6	120.00
Pt1—Pt2—P2	50.31 (3)	C5—C6—H6	120.00
Pt1—Pt2—P3	154.61 (3)	C9—C8—H8	120.00
Br2—Pt2—P2	157.93 (3)	C7—C8—H8	120.00
Br2—Pt2—P3	93.56 (3)	C8—C9—H9	120.00
P2—Pt2—P3	104.41 (4)	C10—C9—H9	120.00
P1—Pt1—H	169.7 (10)	C11—C10—H10	120.00
P2—Pt1—H	85.5 (8)	C9—C10—H10	120.00
Br1A—Pt1—H	77.9 (10)	C10—C11—H11	120.00
Br1B—Pt1—H	89.2 (9)	C12—C11—H11	120.00
Pt2—Pt1—H	35.1 (9)	C11—C12—H12	120.00
Pt1—Pt2—H	35.1 (11)	C7—C12—H12	120.00
Br2—Pt2—H	77.7 (11)	C15—C14—H14	120.00
P2—Pt2—H	85.4 (11)	C13—C14—H14	120.00
P3—Pt2—H	169.6 (12)	C14—C15—H15	120.00
Pt1—P1—C1	109.25 (16)	C16—C15—H15	120.00
C1—P1—C7	106.0 (2)	C17—C16—H16	120.00
Pt1—P1—C7	116.75 (16)	C15—C16—H16	120.00
Pt1—P1—C13	117.50 (16)	C16—C17—H17	120.00
C7—P1—C13	100.6 (2)	C18—C17—H17	120.00
C1—P1—C13	105.6 (2)	C17—C18—H18	120.00
Pt1—P2—Pt2	79.24 (3)	C13—C18—H18	120.00
Pt1—P2—C19	110.91 (15)	C19—C20—H20	120.00
Pt2—P2—C19	123.38 (16)	C21—C20—H20	120.00
Pt2—P2—C25	110.18 (16)	C20—C21—H21	120.00
Pt1—P2—C25	123.60 (15)	C22—C21—H21	120.00
C19—P2—C25	108.3 (2)	C23—C22—H22	120.00
Pt2—P3—C43	115.63 (14)	C21—C22—H22	120.00
C31—P3—C37	105.31 (16)	C24—C23—H23	120.00
Pt2—P3—C37	110.81 (13)	C22—C23—H23	120.00
C37—P3—C43	105.24 (19)	C19—C24—H24	120.00
C31—P3—C43	102.59 (19)	C23—C24—H24	120.00
Pt2—P3—C31	116.14 (15)	C25—C26—H26	120.00
P1—C1—C2	118.0 (4)	C27—C26—H26	120.00
C50—O1—C51	116.5 (8)	C28—C27—H27	120.00
C2—C1—C6	119.5 (4)	C26—C27—H27	120.00
P1—C1—C6	122.5 (4)	C27—C28—H28	120.00
C1—C2—C3	120.3 (5)	C29—C28—H28	120.00
C2—C3—C4	120.1 (5)	C30—C29—H29	120.00
C3—C4—C5	119.7 (5)	C28—C29—H29	120.00
C4—C5—C6	120.8 (5)	C25—C30—H30	120.00
C1—C6—C5	119.5 (4)	C29—C30—H30	120.00
C8—C7—C12	120.0 (5)	C33—C32—H32	120.00
P1—C7—C12	118.9 (4)	C31—C32—H32	120.00
P1—C7—C8	121.1 (4)	C34—C33—H33	120.00
C7—C8—C9	119.8 (6)	C32—C33—H33	120.00
C8—C9—C10	119.9 (6)	C33—C34—H34	120.00
C9—C10—C11	120.1 (6)	C35—C34—H34	120.00
C10—C11—C12	120.1 (6)	C34—C35—H35	120.00
C7—C12—C11	120.0 (5)	C36—C35—H35	120.00
P1—C13—C14	123.6 (4)	C35—C36—H36	120.00
P1—C13—C18	117.2 (4)	C31—C36—H36	120.00
C14—C13—C18	119.2 (5)	C37—C38—H38	120.00
C13—C14—C15	120.3 (5)	C39—C38—H38	120.00
C14—C15—C16	120.4 (5)	C40—C39—H39	120.00
C15—C16—C17	119.8 (5)	C38—C39—H39	120.00
C16—C17—C18	120.2 (5)	C39—C40—H40	120.00
C13—C18—C17	120.2 (5)	C41—C40—H40	120.00
P2—C19—C20	117.3 (3)	C42—C41—H41	120.00
C20—C19—C24	119.2 (4)	C40—C41—H41	120.00
P2—C19—C24	123.1 (4)	C37—C42—H42	120.00
C19—C20—C21	120.2 (4)	C41—C42—H42	120.00
C20—C21—C22	119.8 (5)	C45—C44—H44	120.00
C21—C22—C23	120.7 (6)	C43—C44—H44	120.00
C22—C23—C24	120.4 (6)	C44—C45—H45	120.00
C19—C24—C23	119.6 (5)	C46—C45—H45	120.00
P2—C25—C26	116.9 (3)	C47—C46—H46	120.00
P2—C25—C30	123.7 (4)	C45—C46—H46	120.00
C26—C25—C30	118.9 (4)	C46—C47—H47	120.00
C25—C26—C27	120.4 (5)	C48—C47—H47	120.00
C26—C27—C28	119.9 (5)	C43—C48—H48	120.00
C27—C28—C29	120.4 (5)	C47—C48—H48	120.00
C28—C29—C30	120.8 (5)	O1—C50—C49	110.2 (9)
C25—C30—C29	119.5 (4)	O1—C51—C52	110.7 (9)
C32—C31—C36	119.1 (4)	C50—C49—H49A	110.00
P3—C31—C36	123.5 (3)	C50—C49—H49B	109.00
P3—C31—C32	117.3 (3)	C50—C49—H49C	109.00
C31—C32—C33	120.3 (4)	H49A—C49—H49B	109.00
C32—C33—C34	120.1 (4)	H49A—C49—H49C	109.00
C33—C34—C35	119.9 (4)	H49B—C49—H49C	109.00
C34—C35—C36	120.6 (4)	O1—C50—H50A	110.00
C31—C36—C35	120.0 (4)	O1—C50—H50B	110.00
P3—C37—C38	118.1 (3)	C49—C50—H50A	110.00
C38—C37—C42	119.2 (4)	C49—C50—H50B	110.00
P3—C37—C42	122.7 (3)	H50A—C50—H50B	108.00
C37—C38—C39	120.3 (4)	O1—C51—H51A	109.00
C38—C39—C40	120.4 (5)	O1—C51—H51B	110.00
C39—C40—C41	120.0 (5)	C52—C51—H51A	109.00
C40—C41—C42	120.3 (5)	C52—C51—H51B	109.00
C37—C42—C41	119.9 (4)	H51A—C51—H51B	108.00
C44—C43—C48	118.8 (4)	C51—C52—H52A	109.00
P3—C43—C44	121.5 (3)	C51—C52—H52B	110.00
P3—C43—C48	119.7 (3)	C51—C52—H52C	110.00
C43—C44—C45	120.2 (4)	H52A—C52—H52B	109.00
C44—C45—C46	120.6 (5)	H52A—C52—H52C	109.00
C45—C46—C47	119.7 (5)	H52B—C52—H52C	109.00
C46—C47—C48	120.7 (5)		
			
Br1A—Pt1—Pt2—Br2	29.49 (3)	C43—P3—C31—C32	−63.9 (4)
Br1A—Pt1—Pt2—P2	−165.31 (4)	C31—P3—C37—C42	87.1 (4)
Br1A—Pt1—Pt2—P3	−159.11 (7)	C37—P3—C31—C36	5.2 (4)
P1—Pt1—Pt2—Br2	−168.15 (7)	C31—P3—C43—C44	−15.4 (4)
P1—Pt1—Pt2—P2	−2.95 (8)	C43—P3—C37—C38	159.6 (3)
P1—Pt1—Pt2—P3	3.25 (11)	Pt2—P3—C37—C42	−146.6 (3)
P2—Pt1—Pt2—Br2	−165.20 (4)	Pt2—P3—C43—C48	39.4 (4)
P2—Pt1—Pt2—P3	6.20 (7)	C43—P3—C31—C36	115.1 (4)
Pt2—Pt1—P1—C1	−103.09 (17)	C43—P3—C37—C42	−20.9 (4)
Br1A—Pt1—P1—C1	60.59 (16)	C37—P3—C43—C48	−83.3 (4)
P2—Pt1—P1—C1	−105.44 (16)	Pt2—P3—C31—C36	−117.8 (4)
Pt2—Pt1—P1—C7	136.76 (17)	C31—P3—C43—C48	166.8 (3)
Br1A—Pt1—P1—C7	−59.55 (17)	C50—O1—C51—C52	−170.5 (10)
P2—Pt1—P1—C7	134.41 (17)	C2—C1—C6—C5	1.5 (7)
Pt2—Pt1—P1—C13	17.2 (2)	C51—O1—C50—C49	−179.7 (10)
Br1A—Pt1—P1—C13	−179.16 (18)	C6—C1—C2—C3	−0.8 (8)
P2—Pt1—P1—C13	14.81 (18)	P1—C1—C2—C3	−177.8 (4)
Br1A—Pt1—P2—Pt2	39.94 (10)	P1—C1—C6—C5	178.3 (4)
P1—Pt1—P2—Pt2	178.72 (4)	C1—C2—C3—C4	−1.2 (9)
Pt2—Pt1—P2—C19	−121.79 (17)	C2—C3—C4—C5	2.6 (9)
Br1A—Pt1—P2—C19	−81.85 (19)	C3—C4—C5—C6	−1.9 (9)
P1—Pt1—P2—C19	56.92 (17)	C4—C5—C6—C1	−0.2 (8)
Pt2—Pt1—P2—C25	107.17 (19)	C8—C7—C12—C11	−2.1 (8)
Br1A—Pt1—P2—C25	147.11 (19)	P1—C7—C8—C9	−179.7 (4)
P1—Pt1—P2—C25	−74.11 (19)	P1—C7—C12—C11	179.5 (5)
Br2—Pt2—P2—Pt1	39.20 (9)	C12—C7—C8—C9	2.0 (8)
P3—Pt2—P2—Pt1	−177.26 (3)	C7—C8—C9—C10	0.3 (9)
Pt1—Pt2—P2—C19	108.05 (19)	C8—C9—C10—C11	−2.3 (10)
Br2—Pt2—P2—C19	147.25 (18)	C9—C10—C11—C12	2.2 (11)
P3—Pt2—P2—C19	−69.21 (18)	C10—C11—C12—C7	0.1 (10)
Pt1—Pt2—P2—C25	−122.02 (16)	P1—C13—C14—C15	−177.6 (4)
Br2—Pt2—P2—C25	−82.82 (18)	C14—C13—C18—C17	0.0 (8)
P3—Pt2—P2—C25	60.72 (16)	C18—C13—C14—C15	0.1 (8)
Pt1—Pt2—P3—C31	0.85 (15)	P1—C13—C18—C17	177.9 (4)
Br2—Pt2—P3—C31	172.84 (12)	C13—C14—C15—C16	−0.3 (9)
P2—Pt2—P3—C31	5.77 (13)	C14—C15—C16—C17	0.3 (9)
Pt1—Pt2—P3—C37	−119.22 (14)	C15—C16—C17—C18	−0.2 (9)
Br2—Pt2—P3—C37	52.77 (14)	C16—C17—C18—C13	0.0 (9)
P2—Pt2—P3—C37	−114.30 (14)	P2—C19—C20—C21	−175.3 (4)
Pt1—Pt2—P3—C43	121.17 (16)	C20—C19—C24—C23	2.7 (7)
Br2—Pt2—P3—C43	−66.84 (15)	P2—C19—C24—C23	175.4 (4)
P2—Pt2—P3—C43	126.09 (15)	C24—C19—C20—C21	−2.2 (7)
Pt1—P1—C1—C2	59.8 (4)	C19—C20—C21—C22	1.0 (8)
C7—P1—C1—C2	−173.7 (4)	C20—C21—C22—C23	−0.3 (10)
C13—P1—C1—C2	−67.5 (4)	C21—C22—C23—C24	0.8 (10)
Pt1—P1—C1—C6	−117.1 (4)	C22—C23—C24—C19	−2.1 (9)
C7—P1—C1—C6	9.5 (5)	C26—C25—C30—C29	1.9 (7)
C13—P1—C1—C6	115.6 (4)	C30—C25—C26—C27	0.2 (7)
Pt1—P1—C13—C14	−124.8 (4)	P2—C25—C26—C27	−172.8 (4)
Pt1—P1—C7—C8	12.7 (5)	P2—C25—C30—C29	174.3 (4)
C1—P1—C7—C8	−109.2 (4)	C25—C26—C27—C28	−2.5 (7)
C13—P1—C7—C8	141.0 (4)	C26—C27—C28—C29	2.9 (8)
Pt1—P1—C7—C12	−169.1 (4)	C27—C28—C29—C30	−0.8 (9)
C1—P1—C7—C12	69.1 (5)	C28—C29—C30—C25	−1.5 (8)
C13—P1—C7—C12	−40.7 (5)	C36—C31—C32—C33	0.9 (7)
C7—P1—C13—C14	107.3 (5)	P3—C31—C32—C33	179.9 (4)
Pt1—P1—C13—C18	57.4 (4)	C32—C31—C36—C35	−0.1 (7)
C1—P1—C13—C14	−2.7 (5)	P3—C31—C36—C35	−179.1 (4)
C7—P1—C13—C18	−70.4 (4)	C31—C32—C33—C34	−0.9 (7)
C1—P1—C13—C18	179.6 (4)	C32—C33—C34—C35	0.2 (8)
C25—P2—C19—C20	−154.3 (4)	C33—C34—C35—C36	0.6 (8)
C19—P2—C25—C26	−163.2 (4)	C34—C35—C36—C31	−0.6 (8)
Pt1—P2—C25—C30	156.3 (3)	P3—C37—C38—C39	179.1 (3)
Pt1—P2—C25—C26	−31.1 (4)	C42—C37—C38—C39	−0.4 (6)
Pt1—P2—C19—C20	67.2 (4)	C38—C37—C42—C41	−0.5 (6)
Pt2—P2—C19—C20	−23.6 (4)	P3—C37—C42—C41	−179.9 (4)
Pt2—P2—C25—C26	59.2 (4)	C37—C38—C39—C40	0.9 (7)
Pt1—P2—C19—C24	−105.6 (4)	C38—C39—C40—C41	−0.6 (8)
Pt2—P2—C19—C24	163.7 (3)	C39—C40—C41—C42	−0.3 (8)
Pt2—P2—C25—C30	−113.5 (4)	C40—C41—C42—C37	0.8 (7)
C25—P2—C19—C24	33.0 (5)	P3—C43—C44—C45	−178.3 (4)
C19—P2—C25—C30	24.2 (5)	C44—C43—C48—C47	0.5 (7)
Pt2—P3—C31—C32	63.2 (4)	C48—C43—C44—C45	−0.5 (7)
C37—P3—C31—C32	−173.8 (3)	P3—C43—C48—C47	178.3 (4)
C37—P3—C43—C44	94.5 (4)	C43—C44—C45—C46	−0.2 (8)
Pt2—P3—C43—C44	−142.9 (3)	C44—C45—C46—C47	1.0 (8)
Pt2—P3—C37—C38	34.0 (3)	C45—C46—C47—C48	−1.0 (7)
C31—P3—C37—C38	−92.4 (3)	C46—C47—C48—C43	0.3 (7)

### Hydrogen-bond geometry (Å, º)

Cg4, Cg5 and Cg7 are the centroids of the C13–C18, C19–C24 and C31–C36 rings, respectively.

**Table:**

*D*—H···*A*	*D*—H	H···*A*	*D*···*A*	*D*—H···*A*
C8—H8···Br1*A*	0.95	2.73	3.460 (6)	134
C8—H8···Br1*B*	0.95	1.99	2.719 (13)	132
C45—H45···O1^i^	0.95	2.54	3.484 (8)	170
C48—H48···Br2	0.95	2.65	3.520 (5)	153
C2—H2···*Cg*5	0.95	2.70	3.637 (6)	168
C24—H24···*Cg*4	0.95	2.83	3.459 (9)	125
C30—H30···*Cg*7	0.95	2.84	3.340 (5)	114

Symmetry code: (i) *x*−1, *y*+1, *z*.
